# Analysis of Tribological Properties of Engine Lubricants Used in Hybrid Vehicles

**DOI:** 10.3390/ma17215304

**Published:** 2024-10-31

**Authors:** Daria Skonieczna, Oleksandr Vrublevskyi, Michał Janulin, Piotr Szczyglak

**Affiliations:** Department of Vehicles and Machinery Exploitation, University of Warmia and Mazury in Olsztyn, 10-719 Olsztyn, Poland; aleksander.wroblewski@uwm.edu.pl (O.V.); michal.janulin@uwm.edu.pl (M.J.); szczypio@uwm.edu.pl (P.S.)

**Keywords:** petrol, viscosity, chemical elements, wear

## Abstract

A problem has been noted regarding the admixture of fuel to a low viscosity lubricant in hybrid electric vehicles (HEVs). This is very detrimental to the wear and tear of engine operating components. In this study, the operating conditions of HEVs were analysed. Using X-ray fluorescence spectrometry (XRF), engine oils of two different viscosity classes were compared after the operating process and these data were compared with fresh reference samples. Attention was paid to the content of elements such as Ca, Zn, Mo, Sn, Cd, Fe Pb, Si, Cu, and Ni. The depletion of anti-wear additives, as well as the higher content of metallic wear products relative to the operated 5W30 (engine oil), contributed to the overall assessment of the lubricity of the 0W30 oil, as well as to the tribological results. Then, under laboratory conditions, oil samples contaminated with up to 1 to 8% fuel were subjected to rheological (mini AV-X viscometer) and tribological (four-ball tester) tests. The dependence of the local pressure at the metal-to-metal contact point in the kinematic node on viscosity showed the dissimilar nature of the used and fresh oil and the divergence of the domains for the two groups of samples. Increasing the fuel contamination of used oil above 4% drastically reduces the pressure responsible for maintaining the oil film. In order to improve lubricant performance during HEV operation in urban conditions, it was proposed to carry out extra-urban traffic driving in order to evaporate the fuel from the engine oil. A shorter oil change interval is also recommended.

## 1. Introduction

Today, internal combustion engines are still the most important source of propulsion in transport. This is confirmed by the high percentage of hybrid electric vehicles (HEVs). In view of this, the study of tribological processes and the solution of friction problems associated with the changing requirements and operating conditions of internal combustion engines is a constantly topical issue.

Current literature states that mechanical losses in a passenger vehicle internal combustion engine can absorb up to 60% of the energy gained from fuel combustion [1]. Other sources state that frictional losses in the piston ring assembly can account for 20–30% of the total value [2]. This value is not clear-cut, as it is possible to find works in which authors report the amount of friction loss between piston rings and the cylinder to be 40–70% of the total friction value [3]. This presents a challenge for improving the tribological cooperation of power unit components. Juxtaposing this data with the ever-increasing requirements for exhaust emission levels and environmental concerns, industry and transport are faced with the task of finding an optimum solution to ensure the most efficient lubrication conditions for vehicles and machinery, as well as minimising their negative impact on the environment.

During operation, engine oil is exposed to many factors that contribute to the loss of lubricating properties. One of these is the penetration of contaminants into the engine oil, such as soot [4], for example, whose increased content contributes to accelerated oil degradation. Another factor is the accumulation of wear products. In the literature, it is reported that 50% of vehicles break down due to wear of mating engine components [5]. The lubricating properties of engine oil are also negatively affected by fuel dilution. Due to environmental concerns and the reduction in exhaust emissions, the admixture of ethanol in petrol available at service stations in the European Union is gradually being increased. Unfortunately, the improved environmental conditions can cause dilution of the engine oil and corrosion of the drive unit [6]. The introduction of this additive favours the reduction in CO_2_, NO_x_, hydrocarbons, and particulates [7,8], but this may be at the expense of engine protection.

Both petrol and motor oil are hydrocarbon products. They differ in the viscosity value they have. The permeation of fuel into the engine oil can result in a reduction in the viscosity of the engine oil and thus a deterioration in lubrication conditions due to a reduction in the ability to form and maintain the oil film responsible for fluid film lubrication. Friction between components will increase, leading to worsening mechanical losses and ultimately part damage [9]. Reducing the effectiveness of engine oils by dilution with fuel can result in more than just a reduction in viscosity. Engine conditions in the form of high temperatures and pressures can contribute to undesirable chemical reactions between the fuel and the additives present in the engine oil [10].

The operation of a spark-ignition (SI) engine over short distances can result in fuel accumulation in the lubricant due to the underheating of the drive unit and thus operation on an enriched mixture. Research results show that, in the long term, fuel contamination can be up to 20% [11]. This effect is also influenced by the driver’s driving style and the season in which the vehicle is operated. For the winter months, i.e., operation at lower temperatures, fuel penetration into the engine oil is several percent higher than in the summer months [12]. Reheating the drive unit makes it possible to reduce this impact, however, in the case of hybrid vehicles, where the process of switching the combustion engine on and off automatically is difficult. Another negative effect of fuel entering the lubrication system is to increase the level of oil present in the engine. Assuming a nominal 4 L of oil capacity by the drive unit, a 10% contamination by fuel will increase the liquid volume to 4.4 L. Incomplete combustion of petrol entering the engine oil can also result in sludge formation [13].

Nowadays, with the development of the automotive industry and the increasing popularity of HEVs, the lubricants industry has to meet the requirements for the power units used in these vehicles. For this reason, SAE turned its attention towards lower viscosity index motor oils [14]. In addition to the use of classic motor oil viscosities used in hybrid vehicles such as 5W30 or 0W20, 0W16, and even 0W8 oil viscosities are also encountered. The use of a lubricant with such a low viscosity is intended to protect the drive unit, which is exposed to operation during which it is not fully warmed up (for short distances). Additional dilution with fuel can therefore have major consequences with a direct impact on the condition of the drive unit and its failure.

The engines of modern HEVs, due to the intermittent nature of their operation [15], are exposed to operating with an underheated power unit, which increases the danger of diluting the engine oil with fuel. Studies to show the exposure of fuel to the lubricant in conventional vehicles versus those in hybrid vehicles show that the latter accumulate significantly more fuel [16]. The juxtaposition of this effect with the lack of evaporation due to the lower temperature value of the lubricant operating in hybrid vehicles relative to conventional vehicles poses a major challenge to the development of effective protection for HEV units.

Addressing the issue of fuel contamination in engine oil used in HEVs is due to subjective observations made during oil sampling for laboratory testing. In the course of cooperation with an authorised service station, it was observed that, in the case of some vehicles taken in for periodic scheduled servicing every 15,000 km of mileage, the measured oil level showed an oil level exceeding the maximum value by up to 10 mm. The high percentage of such cases prompted an analysis of the impact of this factor on the protection of the drive unit. Taking into account the hazards mentioned in the literature introduction, which affect lubrication in the engine of a hybrid vehicle, and the authors’ observations in juxtaposition with the relatively new field of motorisation, which is the operation of hybrid vehicles, makes the focus on the phenomenon of engine oil dilution with fuel a topical and very important issue.

The paper consists of five chapters. The first is devoted to a literature review to place the issue under consideration within the existing body of research. The second chapter provides the background to the research in order to highlight the differences in the operation of conventional and HEVs and to draw attention to how lubricant properties change after the operation process. The third section is a description of the subject of the research and the research methods used. The next section is a presentation of the laboratory test results obtained, together with their analysis. The final concluding section brings together all the conclusions that have been drawn from the research conducted. The aim of this study is to determine the maximum fuel admixture that will not endanger the vehicle’s power unit. The utilitarian significance of defining such a limit is that the vehicle user can react to the accumulated contamination and take steps to prevent further fuel accumulation, such as evaporating the excess by driving longer distances or changing the oil.

## 2. Analysis of the Differences in Operating Modes of HEVs and Conventional Combustion Engines

### 2.1. Comparison of Operational Test Results for Conventional and HEV Engines

Differences in the nature of wear and tear on components of conventional internal combustion engines and those used in hybrid engines are presented in this paper [17]. Based on tests of HEVs operated in urban mode, the authors noted that hybrid units showed a higher accumulation of sludge in the engine compartment. At the same time, they drew attention to the fact that the mileage of the hybrid unit was higher compared to the conventional solution. However, the difference in the operating time of the internal combustion engine in the conventional and hybrid solution should be highlighted—in the latter, the operation of the internal combustion engine does not take place throughout the vehicle’s lifetime. Another difference between the lubricants used in conventional engines and hybrid units is the electrical properties: impedance and dielectric strength. Due to this criterion, the friction pairs present in the engine are exposed to damage [18]. From the point of view of the subject under consideration, the key difference between classic vehicle combustion drive solutions and hybrid units is the mode of operation. The authors of the paper [19], on the basis of tests on a Toyota Prius vehicle in the NEDC cycle, recorded 10 starts of the engine, which was switched off 60% of the time during the test. This translates into a significant reduction in lubricant temperature during the operating process. This therefore indicates another discrepancy between the nature of use in the two cases.

Due to the differences in the operating process and its effects for conventional and HEVs described above, a comparative study was carried out to clarify the differences in the operation of these units. The findings presented here are the result of the author’s research and were recorded from the engine control unit.

**Figure 1:**
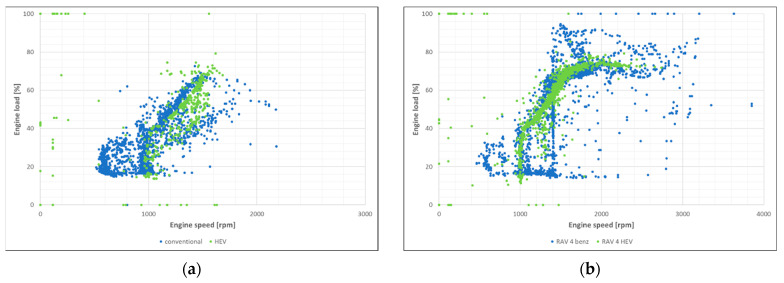
Load map of conventional and HEV engines in urban (**a**) and extra-urban (**b**) modes.

The engine load distribution maps determined during the test in urban and extra-urban modes were determined (Figure 1).

From the data obtained, it can be observed that the operation of the engine in the hybrid drive takes place in a limited load range. For urban mode, speed accumulation occurs in the range of 1000–1500 rpm (conventional system 600–2000 rpm). For extra-urban mode, the HEV operates between 1000 and 2500 rpm (conventional 600–3000 rpm). The loads in urban traffic are in a similar range. In extra-urban traffic, where the HEV engine operates with similar loads, only in a slightly wider speed range, the conventional engine achieves higher speeds and operates with a higher load. A buildup of points at around 1400 rpm can also be observed, which can be explained by non-urban driving at a fixed speed; load variations may be due to the terrain (uphill, downhill, etc.).

**Figure 2:**
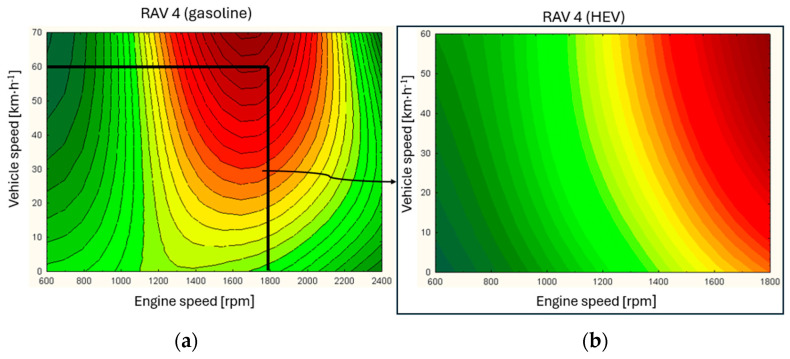
Load characteristics for a conventional engine (**a**) and HEV (**b**) in urban operation.

For the urban mode, the load characteristics were also determined in relation to engine speed and vehicle speed (Figure 2).

Based on the characteristics, it can be concluded that the hybrid unit operates in a limited area of the engine map. The area of highest efficiency and lowest specific fuel consumption is greater for the conventional solution.

By juxtaposing the conventional system with the HEV system in the same range, it can be observed that the load on the combustion engine operating in the hybrid drive unit is distributed diagonally from low vehicle speeds and rotational speeds to high vehicle speeds and analogous rotational speeds. The nature of the load distribution for a conventional unit is different. For low vehicle speeds, the loads in the engine speed range are low to medium. Above 30 km/h the loads take on a full range of values from 0 to above 80%.

**Figure 3:**
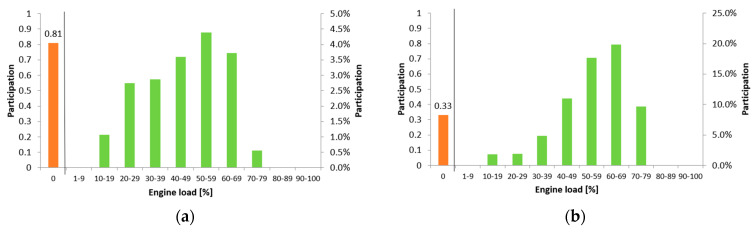
Frequency of HEV combustion engine loads in urban (**a**) and extra-urban (**b**) modes. Orange—idle and engine stop time; green—drive with load corresponding to the range on the x axis.

The proportion of idling and engine off time in urban traffic of a hybrid vehicle is also significantly higher than in non-urban traffic. The difference is as much as 48% (Figure 3).

In urban mode, at low speeds and when the battery is sufficiently charged, the combustion engine can be switched off more often. When the energy stored in the battery is running low, there is a need to recharge the battery, which is realised by switching on the combustion engine. When the battery charge is low, the combustion engine can be used not only to propel the vehicle, but also to charge the battery. This can lead to operation in a less optimal speed range, as the internal combustion engine must simultaneously provide power for propulsion and battery charging. During recuperative braking, the battery is charged by the electric motor acting as a generator. If the battery is almost full, the recuperative braking system may have limited efficiency, as no additional energy will be stored. In winter or extremely hot conditions, the hybrid system may increase the frequency of the internal combustion engine to maintain optimum battery charge and temperature, which affects its efficiency and durability.

**Figure 4:**
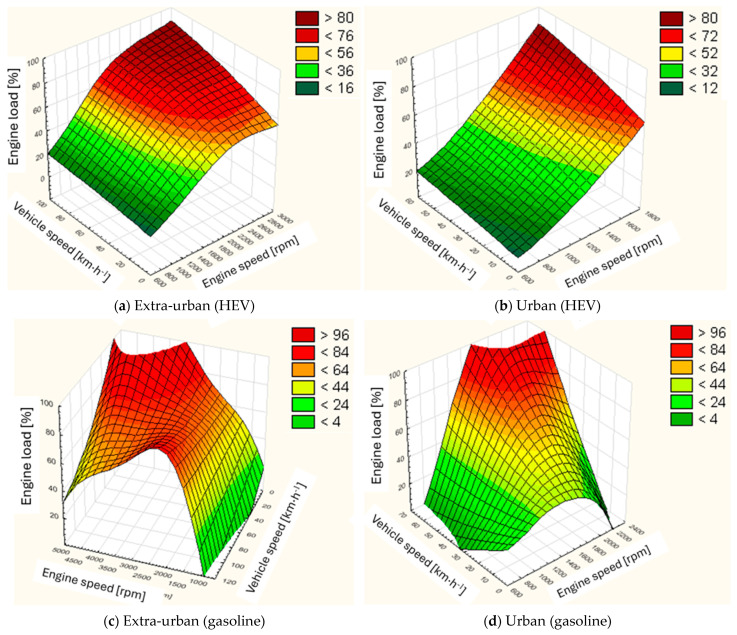
Comparison of load characteristics for conventional and HEV engines in two operating modes.

By juxtaposing the characteristics for the four cases (Figure 4), it can be observed that the widest range of high loads is characterised by the conventional unit used in extra-urban mode (Figure 4c). In urban mode (Figure 4d), due to the higher proportion of low speeds and relatively low engine speeds, the medium load zone dominates. Hybrid vehicles show a different character. Compared to the conventional solution, they have a wider low-load spectrum with a minimum mid-load zone. Juxtaposing the two modes of HEV operation (urban (Figure 4b) and extra-urban (Figure 4a)), it can be seen that the heavy load zone for the extra-urban mode is slightly larger. In addition, the two modes differ in the specifics of load build-up. For the urban mode at low speeds, this build-up is slower and accelerates for higher speeds. In contrast, for the extra-urban mode, the load build-up speed is high at low speeds and slows down at higher speeds.

In summary, the operation of HEVs involves conditions that are different from those of conventional internal combustion engines. The main differences are the speed range and the loads present in the system. A key aspect is the restriction of the area of highest efficiency. Undoubtedly, these differences also affect the process of using lubricants and the possible risk of fuel contamination.

### 2.2. XRF Fluorescence Spectrometric Testing

In the course of vehicle operation, engine components wear out due to friction. This leads to metallic particles entering the lubricant. Information about specific contaminants can be a premise for inferring wear on individual components of the drive unit [20]. Undoubtedly, chemical transformations also occur in the process of lubricant use, which can be caused, for example, by depletion of additives present in the oil, as well as the degradation process itself. These factors affect the quality of protection of the cooperating components.

For this reason, it was decided to supplement the ongoing research with X-ray spectrometry (XRF). The X-MET8000 (Hitachi, Oxfordshire, UK) was used for this part. The equipment used makes it possible to identify the alloy grade or its chemical composition. It is equipped with an X-ray tube and a large-area SDD detector. Each of the four methods carried out (soil, mining, alloy, and precious metals) involved sending X-rays to the sample for 15 s (each sample was tested three times). This resulted in ratios of the elements detected by the instrument contained in the mineral fraction in ppm (soil) and % (mining, alloy, and precious) values. It was decided to use all available methods due to the fact that they complement each other.

Four engine oil samples were tested (two fresh oils: 5W30 and 0W30, as well as the corresponding two used oils). The used oils were from HEVs, with an engine capacity of 2.5 litres. The vehicle mileage for the oil 5W30 was 59,620 km (oil mileage 14,620 km—used in non-urban mode), and for the oil sample 0W30 it was 67,828 (oil mileage 15,987—used in urban mode). The vehicle manufacturer’s declared oil service interval for the selected vehicles was 15,000 km. During the study, results were obtained for the presence of elements such as Ca, Zn, Mo, Sn, Cd, Fe Pb, Si, Cu, and Ni in the samples (Table 1).

Calcium (Ca) in engine oil is a component of detergent/dispersant additives and its key role is to neutralise acidic contaminants [21]. In the case of the 5W30 oil sample, a significant consumption of Ca is noticeable, while for the 0W30 oil its content has only decreased slightly. Another element that is a component of the anti-wear additives for engine oils known as ZDDP (zinc dialkyldithiophosphate) is zinc (Zn) [22]. Results for this element were obtained in all of the four methods carried out. For 5W30 oil, the decrease in the content of this element in the treated sample was at least 21% relative to fresh oil. For 0W30 oil, a slight increase in Zn content relative to the reference sample was achieved in three of the four methods. Increased Zn content in engine oil may also indicate wear in engine internals, such as cams or camshafts. Molybdenum (Mo) can also be classified as an anti-wear additive of particular importance during boundary friction [23]. From the selected test group, a sample of oil in service with a viscosity grade of 5W30 is characterised by a much higher content of Mo compared to the other samples. Such a significant difference may be indicative of the addition of products such as Ceramizer or other similar products to the engine oil to improve lubrication properties and protect the engine.

The group of elements that have their origin in the wear and tear of engine components deserves special attention. When a lubricant does not fulfil its primary function of protecting against metal-to-metal contact, friction occurs, which causes metallic elements to accumulate in the oil. In both cases, an increase in tin (Sn) content was observed for both 5W30 and 0W30 oil. Slip bearings or piston and cylinder components can be the source of this increase in content. One of the key elements that can indicate excessive engine mechanical wear is the iron (Fe) content [7,24]. Iron is the building block of many parts, such as crankshafts, piston rings, or timing gears. In both cases, an almost twofold increase in the content of this element in the lubricant was recorded during the service process. Copper (Cu) is also an element indicative of wear in engine components [24]. Its source can be wear of bearings, bushings, or pins. A significant increase in copper content can also be an indicator of progressive corrosion in the engine compartment. In this aspect, the worse results were obtained with 0W30 oil, as the increase in the content of this contaminant in this case was more than doubled. The 5W30 oil also showed an increase in this contaminant, but not by as much as 5%.

In the case of lead (Pb) content for the samples tested, in both cases, the treated oil samples had a lower lead content than the fresh oil. For oil with a viscosity grade of 5W30, the difference was more than 50%. This may be related to the fact that lead belongs to the heavy element group and thus sinks to the bottom of the oil sump. Silicon (Si) was also found to be lower in used oil than in fresh oil. Its source can be external factors, such as dust, dirt or sand, which enter the engine compartment through leaks [25]. A reduction in its content after the operating process may indicate the effectiveness of filtration in the lubrication system.

An unusual element obtained in the study carried out is cadmium (Cd). Its uniqueness lies in the fact that its source may be external and not necessarily related to the operating process. Contamination of the fuel burned in the chamber with this element, which then enters the lubrication system, may contribute to the elevated cadmium content. It is important to note that cadmium is considered to be a chemical compound that poses a health risk [26]. In the case of 0W30 oil, obtaining a lower content of this contaminant in treated oil relative to fresh oil may be due to the different production batches of the lubricant, in which the reference sample contained a higher percentage of this contaminant. A contaminant whose nature may also be external is nickel (Ni). Its presence in the lubricant may be indicative of wear in high-temperature engine parts, such as valves or camshafts, but it may also accumulate due to a malfunctioning air or fuel intake. In the case of 5W30 oil, the values of used and fresh oil are on a similar level. A difference can be seen in the 0W30 oil, where there is a 0.06% increase in this contaminant in the used oil.

**Figure 5:**
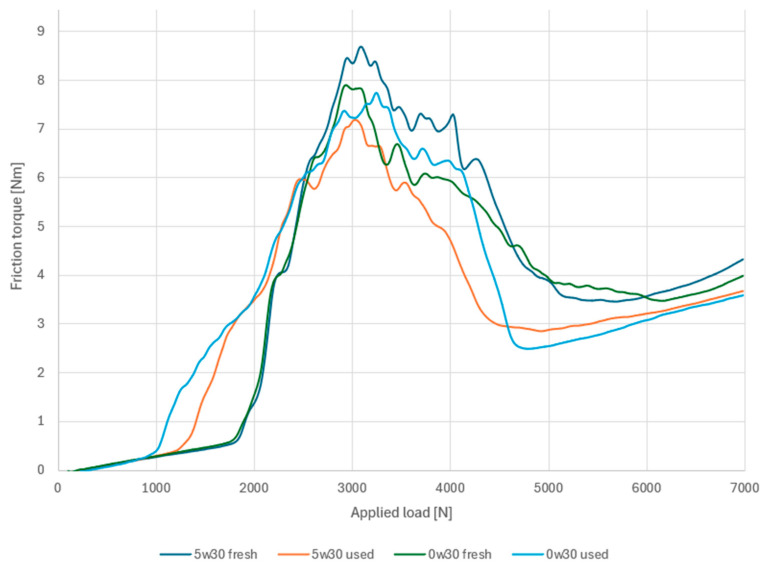
Friction torque diagrams of 5w30 and 0w20 oil samples.

In conclusion, due to the antiwear additives whose components are Ca and Zn, a lower consumption of these additives can be observed with 0W30 oil. The only difference is the molybdenum content, the origin of which was explained during the analysis. With regard to the content of elements whose origin is friction between engine components, the increase in content was comparable (Fe) or greater in the case of 0W30 oil (Cu). In order to draw conclusions about the influence of changes in the content of these elements on operational properties, these should be compared with tribological tests (Figure 5). Tribological tests were carried out according to the methodology described in chapter 3.

In the area of scuffing loads, i.e., the first rapid increase in friction torque, the fresh oils show similar behaviour. The value of this parameter for both samples is 1795 N. Comparing these data with Table 1, in the content of elements present in the anti-wear additives, such as Ca, Zn is higher for the 5W30 oil. The opposite trend, however, occurs with regard to Mo, as its content for the 0W30 sample is about 16 times higher for the test determining the elemental content in ppm. Moving on to the test samples of the oils subjected to the service process, the scuffing load values for the 5W30 and 0W30 oil were 1278 N and 1014 N. Due to the fact that in the case of scuffing load, a wear mark starts to form due to boundary friction, attention should be paid to molybdenum. Its content in used 5W30 is significantly higher, making this oil more load-bearing than 0W30 oil. It should be noted that used 5W30 engine oil has the highest molybdenum content of all the samples tested; however, it does not have the highest scuffing load value. This may be related to its degradation as indicated by the more than doubled calcium content and the increase in the content of the wear elements Fe and Cu.

The increase in the content of wear products, i.e., iron, was comparable for both pairs of fresh and used oil samples. When comparing the used oil samples, the content of engine wear products, i.e., iron, lead, and copper, was higher in the 0W30 sample. In combination with the poorer content of anti-wear additives, this translated into a lower scuffing load and a higher maximum friction torque obtained during the test.

The feature that distinguishes the oil used 5W30 from the other samples is also the shape of the friction torque characteristic. In the case of the other samples, the fluctuation of this parameter was recorded over a wider range of loads. The friction torque of the sample marked in orange on graph 5 began to decrease immediately after reaching the peak value. The peak value of the friction torque corresponds to the end of the kinematic node running-in process. It can therefore be concluded that after the node ran in, an oil film was recreated in the tribosystem, which caused a decrease in the friction torque. Once again, the feature that distinguished this sample is the significant content of molybdenum, which could have contributed to such a reaction of the system to the operating conditions. As can be concluded from the conducted studies, the process of chemical changes is also influenced by the mode of operation. 0W30 oil used in urban mode and thus exposed to uneven operating mode (driving short distances, frequent stops at traffic lights) results in a higher percentage of wear products and thus worse lubricating properties (tribological tests). To sum up, the consumption of the lubricant and its protective properties are undoubtedly influenced by depletion of anti-wear additives and accumulation of wear products from the engine compartment. Due to the fact that the process of operation and wear of structural elements is an individual aspect for each unit, the ratio of the impact of these two aspects is different in each case. Changing proportions of the chemical composition are reflected in the tested samples and their ability to work under variable load conditions.

## 3. Materials and Methods

The subjects of the study were two engine oils. The used oil with a viscosity grade of 5W30 came from a Toyota RAV 4 hybrid vehicle (Table 2) used in mixed mode. The mileage of the vehicle was 67,342 km. The second engine oil sample was fresh oil of the same viscosity grade taken from the resources of the service centre (Olsztyn, Poland) where the vehicle was serviced.

From the data available on the manufacturer’s website [27], the efficiency of the A25A engines used in Toyota RAV 4 conventional systems is 41% and in HEVs is 40%. The D-4S type of fuel supply to the combustion chamber is characterised by the presence of two injections: one direct and one occurring in the intake manifold. This solution ensures the spreading of the fuel mixture throughout the entire volume of the combustion chamber. For low loads and low engine speeds, indirect injection in the intake manifold is used. For medium loads and medium speeds, fuel injection occurs simultaneously in the manifold and cylinder, resulting in increased combustion stability and reduced exhaust emissions. For high loads and engine speeds, only direct injection into the combustion chamber works, which reduces the likelihood of knocking combustion.

The vehicle under consideration uses a hybrid system based on the Toyota Hybrid Synergy Drive (THS) system. It combines the features of series and parallel drive, resulting in efficient use of both the electric motor and the combustion engine. The system’s combustion engine operates on the Atkinson cycle, which improves fuel economy at the expense of a slightly lower maximum power output. When driving in urban mode (at low speed), the vehicle can be driven solely by the electric motor (energy from the battery). When power is required, the system automatically switches between the operation of the internal combustion engine and the electric motor or imposes their simultaneous operation to optimise performance.

On the basis of observations of vehicles using the service, the occurrence of exceeding the maximum oil level read by the dipstick was observed. In extreme cases, the overrun was 10 mm. Using the data obtained, the percentage contamination of engine oil with fuel was estimated. The volume of oil in the lubrication system of the A25A engine is 4.5 L [27].

Under laboratory conditions, oil samples were prepared with a fuel additive (EuroSuper 95 distributed by a local manufacturer—Orlen, Olsztyn, Poland). According to the specification sheet, petrol has a viscosity below 20.5 cSt [28]. Samples ranging from 1 to 8% (in 1% increments) of fuel and control samples without additive were prepared. A magnetic stirrer was used to prepare homogeneous solutions after adding petrol to the oil.

The samples prepared in this way were intended for rheological and tribological tests. Rheological tests included viscosity measurements of the prepared samples. This process took place in two cycles. The first took place immediately after fuel was added to the oil, while the second took place after evaporation of the light fractions. A mini AV-X (Halstreen, The Netherlands) viscometer was used to determine viscosity. The rheologicals were carried out in accordance with EN ISO 3104:1996 and were conducted at 40 °C. The measurement accuracy of the mini AV-X is ±1%.

Tribological tests were carried out using a T-02U four-ball tester (Radom, Poland) [29]. During an 18 s test run, the load applied to a node consisting of three stationary balls and one (top) rotating ball increased linearly at 409 N·s^−1^. Operation of the kinematic node took place at a constant speed of 500 rpm and at an initial temperature of 20 °C. A strain gauge sensor from HBM, type S2M, with an accuracy class of 0.02, was used to acquire friction torque data. As a complement to the study, imaging of the wear marks obtained on the kinematic node was also performed, along with measurements of their diameters. The mass wear of the kinematic node was also determined. The data obtained were intended for further analysis, which is described in the next chapter.

## 4. Test Results

### 4.1. Results of Rheological Tests

**Figure 6:**
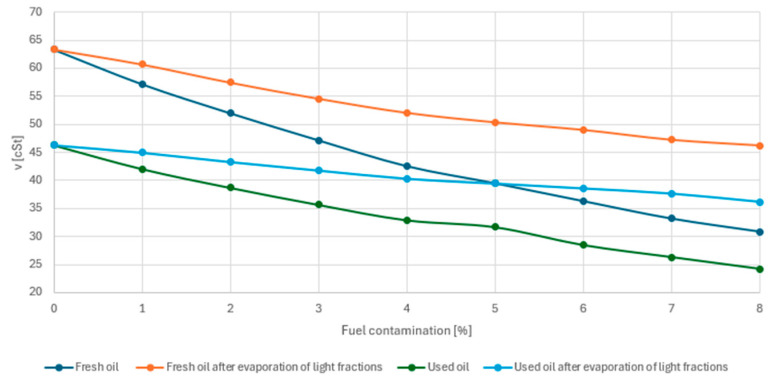
Variation in viscosity as a function of percentage fuel contamination.

As can be seen from Figure 6, the change in viscosity of individual samples as a function of the percentage of petrol contamination including the evaporation process is a non-linear process.

As the fuel dosage increases, viscosity decreases. The changes in viscosity values after the evaporation process of the light petrol fractions proved to be different for fresh and used oil. Despite using the same petrol–oil ratio, the fresh oil sample with petrol admixture showed a greater increase in viscosity after the evaporation process relative to used oil. When comparing with each other, the percentage decrease in viscosity relative to oil without fuel admixture after the evaporation process Δν was also found to be higher for fresh oil. Due to the not directly proportional drop in viscosity with respect to fuel admixture, the additional parameter Δν_fg_ and Δν_ug_ was introduced as the product of the drop in viscosity and the value of fuel admixture.

Table 3 shows a comparison of viscosity changes for fresh and used oil. It is noted that the Δν_fg_ and Δν_ug_ values decrease with increasing contamination. The limits for fresh oil range from 4.21 to 3.38 and for used oil from 2.89 to 2.74. At this stage, therefore, a partial conclusion can be drawn that used oil contaminated with fuel retains its rheological properties more stably than fresh oil. The changes taking place in fresh oil cover a wider range of viscosity values and result in a steeper drop in the value of this parameter.

### 4.2. Tribological Test Results

**Figure 7:**
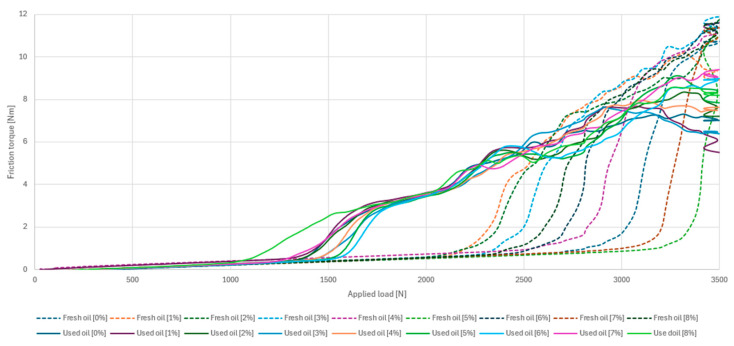
Characteristics of the friction torque depending on the applied load.

All samples contaminated with fuel and samples free of this contamination were subjected to tribological tests. For the fresh oil group, the maximum allowable friction torque of 10 Nm was exceeded for eight of the nine samples. The sample contaminated with 1% petrol was close to reaching the critical threshold of frictional torque generated at the friction node; however, in this case the machine did not stop. In the case of the used oil samples, one sample also deviated in its characteristics. Contamination of the oil with 7% petrol led to the node seizing and exceeding the limiting friction torque. The largest differences were observed for the samples in the P_t_ scuffing load area (Figure 7).

Despite the admixture of petrol and the lower scuffing load value, the used oil showed greater stability. The range of P_t_ scuffing loads was in the range 1048–1548 N. For fresh oil, the range was twice as large, ranging from 2134 to 3177 N. The accuracy of determining the scuffing load P_t_ is 1 N.

**Figure 8:**
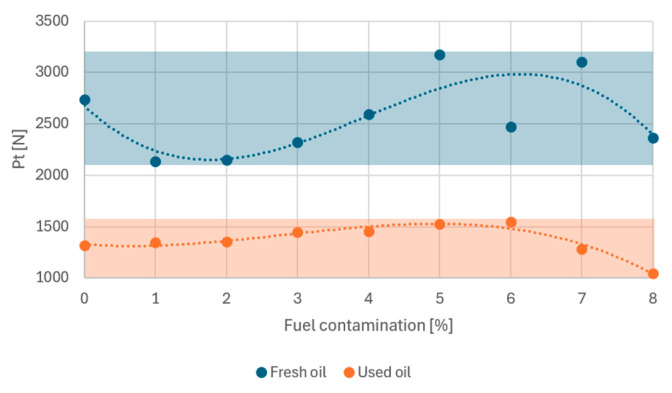
Scuffing load depending on fuel contamination.

Small additions of fuel to the treated oil caused the P_t_ value to increase until a breakthrough of 6% was reached, followed by a decrease in this value (Figure 8). A similar phenomenon was observed for fresh oil. Fuel contamination of 1% resulted in a significant drop in P_t_ relative to the ‘0’ sample; however, increasing contamination to 5% resulted in higher scuffing load values. The fresh oil sample with 6% fuel contamination would have to be rejected due to the large discrepancy in its values relative to neighbouring samples.

Note also the area occupied by the recorded P_t_ values for both groups of samples, marked on the graph as a blue and orange rectangle. For fresh oil, this area is about twice as large, indicating a greater spread of values and less stability to fuel addition.

At this stage, a partial conclusion can be drawn that, in the area of mashing loads, the operated oil is more stable towards the start of the mashing process than fresh oil. From an operational point of view, this is advantageous, as the process of contamination of the oil with fuel does not occur rapidly. For both groups, a breakthrough point of 6% fuel contamination can be identified, which results in a significant deterioration of the anti-friction properties. In the remainder of this work, this point will be verified as the limiting point at which the lubricating properties of the engine oil are lost.

**Figure 9:**
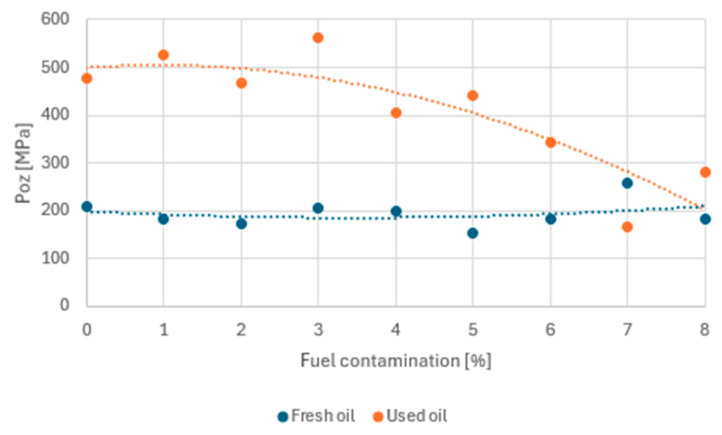
Seizing pressure limit in relation to fuel contamination.

The limiting seizure load P_oz_ was also determined (with precision 0.01 MPa) for the samples tested (Figure 9), defined as:

P_oz_ = 0.52·p_oz_·d^−2^ (1)

where p_oz_ is the limiting seizure load [N] and d is the average diameter of the wear mark on the stationary balls [mm]. In this case, the used oil showed higher values for this parameter and less stability against fuel contamination. There is a critical drop in the limiting seizure load P_oz_ around 5–6% of fuel in the used oil. Fresh oil does not show such a drop and retains P_oz_ values in a stable range regardless of fuel contamination.

**Figure 10:**
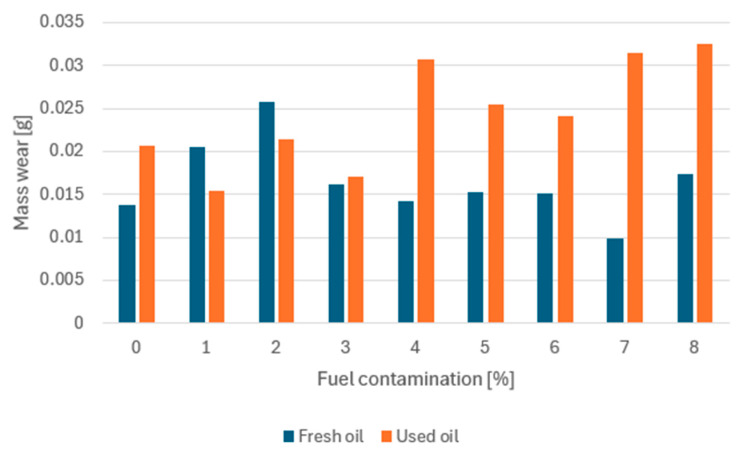
Massive wear of elements of the kinematic node.

From an exploitation point of view, the primary function of lubricating oil is to protect the mating components. This protection consists of creating an oil film and maintaining it so that metal-to-metal contact is impossible, i.e., maintaining the conditions of fluid friction. If this condition is not fulfilled and the oil film breaks, boundary friction and wear of the system components occurs. To supplement the results obtained, an attempt was made to determine the mass wear of the four balls comprising the kinematic node of the T-02U tester (Figure 10). The mass of the kinematic node before the test was 33.4181 g; the measurement accuracy of the scale is 0.0001 g. However, by comparing the data obtained for all samples, some differences can be observed.

For used oil, mass consumption intensifies once 4% fuel contamination is exceeded. For fresh oil, the highest mass loss was recorded for a sample contaminated with 2% fuel.

In summary, the tests carried out indicate that an assumed 6% fuel contamination is the stage at, which an intensification of the consumption process was recorded for used oil. Therefore, 4% should be taken as a safe limit for fuel admixture to oil. In the case of fresh oil, an intensification of mass consumption occurred for a contamination of 2–3%. These data are the same as the results obtained for mash loads (Figure 8). A reduction in P_t_ contributed to an increase in the mass consumption of the samples.

### 4.3. Importance of Fuel Admixtures to Oil Under Operating Conditions

As is well known, several key criteria are required to maintain the oil film in an engine. These include viscosity and oil pressure. The results obtained from research indicate that fuel contamination significantly reduces lubricant viscosity. Considering the issue more broadly and taking into account the fact that viscosity decreases during operation due to the breaking up of longer hydrocarbon chains [30], oil subjected to operation for this reason immediately has a lower viscosity compared to fresh oil. Fuel admixture in such a situation can contribute to reaching a critical point beyond which the mating components are no longer effectively protected. 

**Figure 11:**
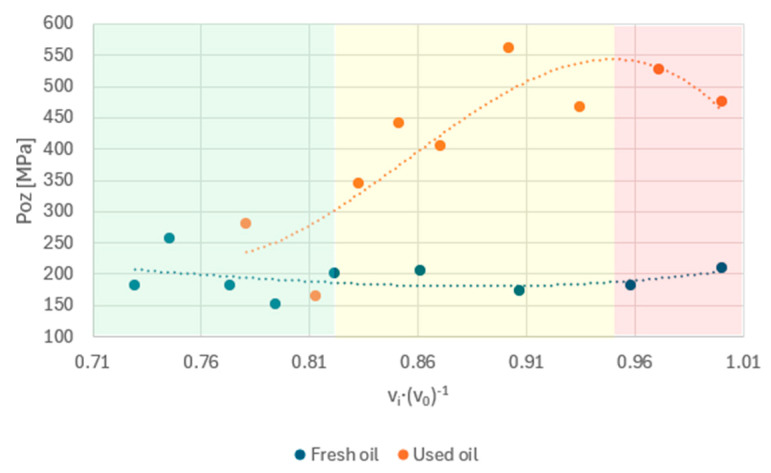
Variation in oil film pressure as a function of viscosity v_i_·(v_0_)^−1^.

The nature of the effect of the addition of fuel to the engine oil on the pressure at the ball contact point differs between fresh and used oil (Figure 11).

Analysing the data in the relative viscosity space v_i_·(v_0_)^−1^, fresh oil retains similar values of the P_oz_ parameter over the entire range of fuel admixture changes despite viscosity changes. Exposed oil located in the lower viscosity range shows variable characteristics. From the results obtained, it can therefore be interpreted that during oil operation, the pressures occurring at the point of contact between the components decrease with fuel dilution. The area of this characteristic can be divided into three sections, which are marked on the graph. The green area is the range in which the properties of fresh oil and used oil are similar. In the yellow area, fresh oil shows stability in its properties, while used oil shows a significant change in the P_oz_ parameter in terms of relative viscosity. In the red area, i.e., at the lowest fuel contamination rates, the oils show different characteristics. In the case of used oil, as the fuel dose decreases, the value of the contact pressure between the balls decreases, while fresh oil shows an increase in this parameter.

**Figure 12:**
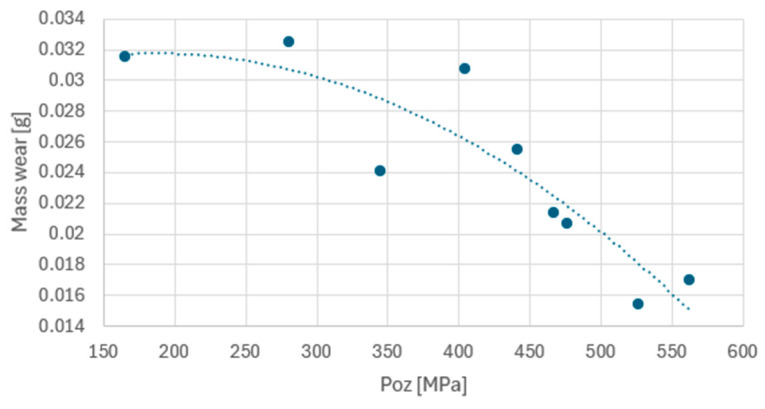
Dependence of pressure at the contact point of the mass wear.

In order to refer to the wear processes, the obtained values of the P_oz_ parameter were compared with the mass consumption data for the oil undergoing the wear process (Figure 12). With regard to the fuel contamination cut-off point proposed earlier in the paper, the 4% fuel admixture indicated is reasonable, as the parabola of the trend line shows the greatest intensification at this point.

As the pressure decreases, the mass wear of the mating components progresses. This relationship is parabolic in nature. It should also be noted that a 25% decrease in the ratio of viscosity obtained after fuel admixture relative to the initial viscosity for the treated oil puts the obtained P_oz_ pressures in the same area as the values obtained for fresh oil, but the mass consumption (Figure 10) is significantly higher for the used oil samples.

## 5. Discussion

The authors of the paper [31] determined that the addition of a 6% blend of bioethanol and petrol to the engine oil resulted in a decrease in viscosity of about 30%. However, it should be noted that the rheological properties of the base sample were higher compared to the samples tested in this paper. The viscosity value of the base sample of the authors of the paper [31] ν = 82.03 cSt, while in the present study, the value was 63.35 cSt, respectively. With a contamination of 6% of the fuel mixture, a viscosity drop of 22.68% was obtained. This confirms the conclusion obtained in this study that the higher the base viscosity of the engine oil, the steeper the drop in viscosity of the engine oil. This is related to the difference in this parameter between fuel and oil. Directing their attention towards the quality of the engine oil in relation to tribological tests, the authors considered the previously mentioned 6% fuel contamination to be the limiting value. The studies included in this paper argue against this limit, as 4% was considered a safe limit. Once again, this is a consequence of the initial viscosity values of the samples under consideration and the subsequent ability to form and maintain an oil film. It can therefore be concluded that, as the manufacturer’s recommended viscosity of the engine oil used is reduced, its sensitivity to fuel admixtures is increased. This is particularly important in hybrid vehicles, which are designed to run on low-viscosity-grade oils due to their operating characteristics.

The problem of oil dilution with fuel does not only apply to spark-ignition engines. The paper [32] indicates that 5–8% contamination of engine oil with diesel fuel should be a premise for corrective action (e.g., oil change). The results obtained by the authors of this paper coincide with the conclusions obtained in this paper, despite the fact that the viscosity of the oil sample considered in paper [32] was significantly higher (105.01 cSt).

Undoubtedly, the condition and properties of the lubricant, as well as the system in which it is located, depend on many factors. It should be noted that the use of engines is not limited to the automotive industry. Their use and the understanding of operation in the wider industry can be beneficial in preventing failures. To this end, it is worth extending the analysis to include modelling of the lubrication system, an example of which is described in the paper [33].

## 6. Conclusions

On the basis of the tests carried out, it was observed that for the same drive unit used in a conventional drive train, there is significantly less admixture to the engine oil of the fuel than is the case with a hybrid system. This is due, among other things, to the fact that the HEV drive unit operates in a reduced load range relative to the conventional unit. This is particularly evident in the load characteristics of both powertrains in extra-urban traffic. The conventional units showed a much wider range of high loads relative to the HEV units, which influences the lower operating temperatures of the engine in the hybrid systems and, consequently, the lower evaporation capacity of the fuel that entered the engine oil. The juxtaposition of the load characteristics against engine speed and vehicle speed in urban traffic conditions makes it possible to conclude that the HEV unit operates in the limited space of the engine map.

(1) The higher load on the drive unit in extra-urban traffic is also evidenced by the results of the analysis of the chemical composition of the oils, from which a faster depletion of the anti-wear additives in extra-urban traffic can be observed. In addition, as a result of comparing the chemical composition of oils subjected to service with different viscosity classes, an increased molybdenum content (by more than 10 times) was noted in the 5W30 oil relative to the 0W30 oil, indicating more efficient generation and maintenance of the oil film in the area of mashing loads and in the area of maximum friction torque. However, this is not an indicator of the overall effectiveness of the engine oil, as this aspect would also need to be analysed based on the content of elements such as Ca (calcium) and Zn (zinc). The overall assessment of the lubricity of the 0W30 oil, as well as the results of tribological tests, were affected by the depletion of anti-wear additives, as well as a higher content of metallic wear products relative to the 5W30 in use.
(2) A study of the effect of fuel admixture on the characteristics and condition of engine oils was also conducted. It was observed that the nature of viscosity changes in engine oil depends on whether one is dealing with fresh or used oil. Viscosity changes caused by fuel dilution in the case of used oil are characterised by a smaller amplitude than is the case with fresh oil. For fresh oil, the decrease in viscosity at the highest fuel admixture compared to a non-fuel-diluted sample was 27.07%, while the corresponding value for used oil was 21.92%.
(3) The operated oil group contaminated with fuel showed a smaller spread of scuffing load values Pt (1048–1548 N) than the fresh oil group (P_t_ 2134–3177 N). At the same time, the junction obliteration process in the case of the operated oil took place in a lower load range than for the fresh oils. The limiting seizure pressure Poz determined for each sample was at a similar level (around 200 MPa) for fresh oil depending on the percentage of fuel contamination and was lower than the non-linear characteristic determined for treated oil.
(4) In addition, increasing the fuel contamination of the used oil drastically reduces the pressure responsible for maintaining the oil film, and this in turn determines the increase in mass consumption. On the basis of the measurements carried out, it was observed that, once fuel contamination exceeds 5%, there is an intensification of mass consumption, which undoubtedly indicates accelerated wear of the drive unit.
(5) Therefore, in view of the varying driving modes, rheological parameters and lower ambient temperatures hindering the evaporation of petrol from the engine oil, a value of 4% should be taken as an acceptable limit for fuel contamination. In view of the above, it should be assumed that when operating HEVs in urban conditions and at ambient temperatures that make it difficult to reach the operating temperature of the power unit, it is advisable to carry out extra-urban driving at higher speeds in order to evaporate the fuel from the engine oil and thus improve the lubricant performance, or to adopt a shorter oil change interval than recommended by the manufacturer.

## Figures and Tables

**Table 1:** Content of selected elements in engine oil samples.

Element	5W30 (Fresh)	Precision	5W30 (Used)	Precision	0W30 (Fresh)	Precision	0W30 (Used)	Precision
			Method: soil					
Ca [ppm]	16,892.00	138.00	8821.50	112.33	8353.67	97.33	7415.33	91.67
Zn [ppm]	5369.33	27.00	4158.50	24.33	4234.00	23.00	4004.33	22.67
Mo [ppm]	12.33	2.00	1500.00	4.67	193.67	3.00	165.00	3.00
Sn [ppm]	38.67	9.00	44.00	9.00	41.00	9.00	45.00	8.00
Cd [ppm]	16.33	7.00	28.50	7.00	17.33	7.00	14.33	6.00
			Method: mining					
Ca [%]	22.24	0.19	14.42	0.18	15.57	0.19	15.02	0.19
Zn [%]	13.70	0.25	10.81	0.24	13.96	0.28	14.42	0.30
Sn [%]	0.80	0.18	1.31	0.23	1.067	0.27	1.54	0.30
Cd [%]	0.43	0.11	0.62	0.13	0.64	0.16	0.61	0.18
			Method: alloy					
Zn [%]	32.36	0.30	23.86	0.27	30.28	0.31	31.02	0.33
Mo [%]	0.28	0.06	18.65	0.11	2.97	0.08	2.89	0.09
Fe [%]	0.57	0.13	0.95	0.14	0.72	0.15	1.47	0.19
Pb [%]	0.32	0.04	0.14	0.03	0.26	0.03	0.22	0.03
Si [%]	0.99	0.14	0.79	0.13	0.73	0.14	0.70	0.16
			Method: precious metals					
Zn [%]	42.03	0.21	26.14	0.17	34.08	0.19	34.09	0.19
Mo [%]	0.43	0.04	21.33	0.07	3.54	0.05	3.18	0.05
Fe [%]	0.74	0.36	1.28	0.18	0.81	0.10	1.65	0.11
Cu [%]	0.12	0.03	0.18	0.03	0.14	0.03	0.36	0.03
Ni [%]	0.14	0.03	0.12	0.03	0.17	0.04	0.24	0.04

**Table 2:** Selected specification data of the HEV Toyota RAV 4–A25A engine [27].

Toyota RAV 4–A25A Engine	
Displacement	2494 cm^3^
Engine type	SI Atkinson cycle
Drive type	hybrid
Engine power	176 HP/163 kW (5700 rpm)
Maximum torque	221 Nm (3600–5200 rpm)
Number of cylinders	4
Cylinder system	in-line
Number of valves	16
Cylinder diameter × piston stroke	87.5 × 103.4 mm
Injection system	Combined injection D-4S (dual)

**Table 3:** Percentage decrease in viscosity of tested samples.

	Fresh Oil			Used Oil		
Fuel Contamination [%]	Viscosity Increase After Evaporation [cSt]	Δν_f_ [%]	Δν_fg_ [-]	Viscosity Increase After Evaporation [cSt]	Δν_u_ [%]	Δν_ug_ [-]
0	-	-	-	-	-	-
1	3.52	4.21	4.21	2.39	2.89	2.89
2	5.46	9.34	4.67	4.62	6.51	3.26
3	7.41	13.89	4.63	6.14	8.82	3.27
4	9.50	17.87	4.47	7.43	12.99	3.25
5	10.86	20.55	4.11	7.79	14.87	2.97
6	12.69	22.68	3.78	10.12	16.74	2.79
7	14.04	25.47	3.64	11.35	18.73	2.68
8	15.36	27.07	3.38	11.97	21.92	2.74

## Data Availability

The original contributions presented in the study are included in the article, further inquiries can be directed to the corresponding author.
